# Vascular endothelial growth factor blockade alters magnetic resonance imaging biomarkers of vascular function and decreases barrier permeability in a rat model of lung cancer brain metastasis

**DOI:** 10.1186/2045-8118-12-5

**Published:** 2015-02-17

**Authors:** Gregory L Pishko, Leslie L Muldoon, Michael A Pagel, Daniel L Schwartz, Edward A Neuwelt

**Affiliations:** Department of Neurology, Oregon Health & Science University, 3181 Sam Jackson Park Road, L603, Portland, OR 97239-3011 USA; Department of Neurosurgery, Oregon Health & Science University, 3181 Sam Jackson Park Road, L603, Portland, OR 97239-3011 USA; Office of Research and Development, Department of Veterans Affairs Medical Center, Portland, OR USA

**Keywords:** Blood–brain barrier, Bevacizumab, Magnetic resonance imaging, Tumor model, Cerebral blood volume, Vascular normalization, Anti-angiogenic drugs, Drug delivery

## Abstract

**Background:**

Blockade of vascular endothelial growth factor (VEGF) to promote vascular normalization and inhibit angiogenesis has been proposed for the treatment of brain metastases; however, vascular normalization has not been well-characterized in this disease. We investigated the effect of treatment with bevacizumab anti-VEGF antibody on magnetic resonance imaging (MRI) biomarkers of brain tumor vascular characteristics in comparison to small molecule delivery in a rat model of human lung cancer brain metastasis.

**Methods:**

Athymic rats with A549 human lung adenocarcinoma intracerebral xenografts underwent MRI at 11.75 T before and one day after treatment with bevacizumab (n = 8) or saline control (n = 8) to evaluate tumor volume, free water content (edema), blood volume and vascular permeability (K^trans^). One day later, permeability to ^14^C-aminoisobutyric acid (AIB) was measured in tumor and brain to assess the penetration of a small drug-like molecule.

**Results:**

In saline control animals, tumor volume, edema and permeability increased over the two day assessment period. Compared to controls, bevacizumab treatment slowed the rate of tumor growth (*P* = 0.003) and blocked the increase in edema (*P* = 0.033), but did not alter tumor blood volume. Bevacizumab also significantly reduced K^trans^ (*P* = 0.033) and AIB passive permeability in tumor (*P* = 0.04), but not to peritumoral tissue or normal brain. Post-treatment K^trans^ correlated with AIB levels in the bevacizumab-treated rats but not in the saline controls.

**Conclusions:**

The correlation of an MRI biomarker for decreased vascular permeability with decreased AIB concentration in tumor after antiangiogenic treatment suggests that bevacizumab partially restored the normal low permeability characteristics of the blood–brain barrier in a model of human lung cancer brain metastasis.

## Background

Brain metastasis occurs in 15–20% of patients with non-small cell lung cancer (NSCLC), resulting in high morbidity and rapid mortality [1]. Current treatments for brain metastases include surgery, whole brain irradiation, and stereotactic radiosurgery [2]. Chemotherapy regimens can show efficacy in brain metastases but typically are less effective than in the systemic mass, at least in part because drug delivery is limited by malformed neovasculature and inconsistent permeability of the blood–brain barrier (BBB) and blood-tumor barrier (BTB) [3]. Treatments for lung cancer brain metastases have only short-term efficacy, and after recurrence there is no standard second-line regimen that offers consistent benefit.

Vascular endothelial growth factor (VEGF) is highly expressed in many human brain tumors [4], where it promotes tumor angiogenesis, providing critical support for tumor growth and survival [5, 6]. Bevacizumab is an anti-VEGF-A monoclonal antibody that inhibits angiogenesis and also promotes vascular normalization by pruning immature vessels and improving perivascular cell and basement membrane coverage and function [7]. As a salvage therapy in progressive malignant glioblastoma, bevacizumab decreases tumor growth and reduces edema and steroid use [8, 9], but recent reports indicate no survival benefit in newly diagnosed glioblastoma [10]. In NSCLC brain metastases, bevacizumab has been proposed as both front-line treatment and as salvage therapy in combination with chemotherapy [11–13].

Magnetic resonance imaging (MRI) techniques provide a non-invasive mechanism to assess tumor vasculature and the effects of bevacizumab over time [14]. We used MRI to measure brain tumor growth, water content (edema) [15, 16], relative cerebral blood volume (rCBV) [17–19], and vascular permeability as determined by the vascular transfer coefficient (K^trans^) [20, 21] in a rat model of human lung cancer brain metastasis. In contrast to the current hypothesis that vascular normalization improves chemotherapy delivery, we hypothesize that restored BBB function will actually decrease drug delivery [22]. The purpose of this study was to determine the effects of bevacizumab on MRI biomarkers of vascular characteristics in comparison to small molecule delivery in brain metastases.

## Methods

### Tumor implantation and treatments

The care and use of animals was approved by the Institutional Animal Care and Use Committee and was supervised by the Oregon Health & Science University (OHSU) Department of Comparative Medicine. The A549 human lung adenocarcinoma cells, obtained from ATCC (American Type Culture Collection, Manassas VA, USA) and used at an early passage number, were cultured in DME with 10% serum and penicillin, streptomycin and gentamicin antibiotics. Adult female nude rats (200–220 g) from the OHSU colony were anesthetized with intraperitoneal (IP) ketamine (60 mg/kg) and diazepam (7.5 mg/kg). Tumor cells (12 μl, ~10^6^ cells, >90% viability) were inoculated at stereotactic coordinates for intracerebral localization in the right caudate putamen (vertical bregma, −3.1 mm lateral, −6.5 mm depth). After tumors developed to >6 mm^3^ (3–4 weeks) rats underwent pretreatment MRI and were randomized 24 h later to receive either 1) intravenous (IV) saline (saline control group) or 2) bevacizumab (Avastin, Genentech, San Francisco, USA, 45 mg/kg IV, n = 8 per group). Post-treatment MRI was performed 24 h after treatment. Aminoisobutyric acid (AIB) passive permeability was assessed 24 h after the post-treatment MRI (48 h after bevacizumab).

### Magnetic resonance imaging

Animals were anesthetized using IP ketamine (60 mg/kg IP) and dexmedetomidine (0.6 mg/kg IP; Henry Schein Animal Health, Dublin OH, USA**)**, with atipamezole (1 mg IP; Henry Schein Animal Health, Dublin OH, USA**)** reversal at end of study**.** Warm air was circulated through the bore of the MRI scanner to maintain a physiological temperature. MRI was performed at 11.75 T (Bruker Corporation, Billerica MA, USA) at the Advanced Imaging Research Center using a Bruker volume coil for transmitting and a Bruker surface coil for receiving. All images were obtained in the axial plane at 1 mm slice thickness. Prior to the injection of contrast agent, anatomical images were acquired: high resolution T_1_-weighted fast low-angle shot (FLASH) (TR/TE = 160.0/1.6 ms; flip angle (FA) = 60°; matrix size = 256 × 256; slices = 25; field of view (FOV) = 3.2 × 3.2 × 2.5 cm; number of averages = 4; TA = 2.72 min); T_2_-weighted rapid acquisition refocusing echoes (RARE) (TR/TE = 4020.6/23.6 ms; RARE factor = 8; matrix size = 256 × 256; slices = 25; FOV = 3.2 × 3.2 × 2.5 cm; number of averages = 1; TA = 2.13 min); and T_2_*-weighted FLASH sequence (TR/TE = 430.3/6.6 ms; FA = 30°; matrix size = 384 × 384; slices = 25; FOV = 3.2 × 3.2 × 2.5 cm; total acquisition time = 2.75 min).

Ferumoxytol (Feraheme, AMAG Pharmaceutical, Inc., Waltham MA, USA), an iron oxide nanoparticle, was delivered by bolus injection (30 mg/ml, 60 μL) via tail vein catheter (3 ml/min). Pre- and post- ferumoxytol high resolution T_2_*-weighted images were acquired for the creation of steady-state ΔR_2_* maps.

Pre-gadolinium based contrast agent (GBCA) longitudinal relaxivity (R_1_) values were acquired by a fast spin-echo inversion recovery sequence (TR/TE = 9000/5.1 ms; inversion times (TI) = 0.14/0.3/0.6/1.2/2.5/5.0/8.5 s; RARE factor = 8; matrix size = 128 × 64; slices = 1; field of view = 4.48 × 2.24 × 0.1 cm; TA = 10.23 min). The same slice was acquired as in the ferumoxytol steady-state MRI. A dynamic contrast-enhanced (DCE)-MRI FLASH sequence (TR/TE = 25.0/1.4 ms; FA = 20°; matrix = 128 × 64; slices = 3; field of view = 4.48 × 2.24 × 0.3 cm; intersampling interval = 1.6 s; repetitions = 400; number of averages = 1; total acquisition time = 10.67 min) was used to track extravasation and washout of low molecular weight gadodiamide, Gd-DTPA-BMA (Omniscan, GE Healthcare, Piscataway, NJ USA), in tumor for ~10 min. Gd-DTPA-BMA was delivered by bolus injection (287 mg/ml, 60 μL) via a tail vein catheter (1 ml/min). Post Gd-DTPA-BMA T_1_-weighted images were acquired after DCE-MRI.

### MRI data analysis

T_2_-weighted images were used to identify tumor perimeter determined by hyperintensity compared to normal surrounding grey matter. Total tumor volume was measured by the summation of tumor volumes in multiple T_2_-weighted slices, manually outlined using ImageJ software (NIH).

MRI-derived measurements of tumor volume, R_1,_ K^trans^, and rCBV were performed before and after treatment and were normalized to percent change from baseline ([post-pre]/pre). All MRI-derived measurements were averaged over voxels within a region of interest (ROI) matched to the central slice used to calculate tumor volume in T_2_-weighted images.

Pre-GBCA R_1_ (R_1_ = 1/T_1_) spin–lattice relaxation rates in the tumor were calculated by fitting MR signal to an inversion recovery model with three fitted parameters: R_1_, M_0_ (Boltzmann magnetization), and α (imperfect inversion correction): S(TI) = │M_0_*(1 – α*exp(−TI*R_1_)│; where S is the MR signal, which is a function of TI.

Vascular permeability parameters were determined using DCE-MRI data modeled with an in-house software (BOLERO) in MATLAB (The Mathworks, Inc., Natick, MA, USA), using a two-compartment model [21] of transvascular exchange between vascular and tissue space to extract permeability as measured by the rate transfer constant, K^trans^ and extravascular-extracellular volume fraction, v_e_. The area under the longitudinal relaxivity R_1_ curve within the tumor is proportional to the concentration of Gd-DTPA in tumor tissue. The arterial input function for the model was measured as MR signal time course of contrast agent in blood in the sagittal sinus during the GBCA injection. The amplitude of the clearance was adjusted for each rat based on a reference tissue (temporalis) method [19].

Blood volume was assessed using steady-state ΔR_2_*, with ferumoxytol as a blood-pool contrast agent. ΔR_2_* maps were created assuming that the changes in transverse relaxation rate have a linear relationship with ferumoxytol contrast agent concentration [23]. Steady-state-CBV maps were created on a pixel-by-pixel basis in MATLAB using the relationship: ΔR_2_* = ln(S_pre_/S_post_)/TE, where S_pre_ is the signal intensity pre-ferumoxytol and S_post_ is the signal intensity at a steady-state signal intensity corresponding to a constant concentration of ferumoxytol in blood. Relative cerebral blood volume (rCBV) was determined by comparing tumor ΔR_2_* to an equivalent ROI in contralateral normal brain.

### Small molecule permeability

The day after post-treatment dynamic MRI, rats received 12.5 μCi of ^14^C-AIB (MW = 103 Da; American Radiolabeled Chemicals, St. Louis MO, USA) administered IV for assessment of unidirectional tracer leakage from the blood to brain tissue. A serum sample was collected 10 min after AIB administration followed by perfusion to clear the vasculature. The brains were harvested and four regions were dissected: tumor mass, tissue 1–2 mm brain around tumor (BAT), tissue 1–2 mm ipsilateral brain distant to tumor (BDT), and contralateral left hemisphere (LH) normal brain tissue. Each tissue sample was weighed, solubilized in 2 ml of sodium hydroxide overnight (MP Biomedicals, Solon, OH, USA) and radioactivity was determined by liquid scintillation counting in 20 ml of CytoScint ES (MP Biomedicals, Solon, OH, USA). Results were measured as percent injected dose normalized to 1 g of tissue.

### Statistical analysis

A Student’s 2-tailed, paired *t*-test was used to test statistical significance of change from pre- to post-treatment for tumor growth, R_1_, K^trans^, v_e_, and steady-state rCBV. Unpaired t-tests were used to assess changes in these parameters comparing the saline control and bevacizumab treatment groups. Pearson’s r was used to measure the strength of the relationship between parameters. Statistical tests were performed using Microsoft Excel and Graphpad Prism. Data within figures are presented as mean and standard error of the mean (SEM) of the change from baseline.

## Results

**Figure 1:**
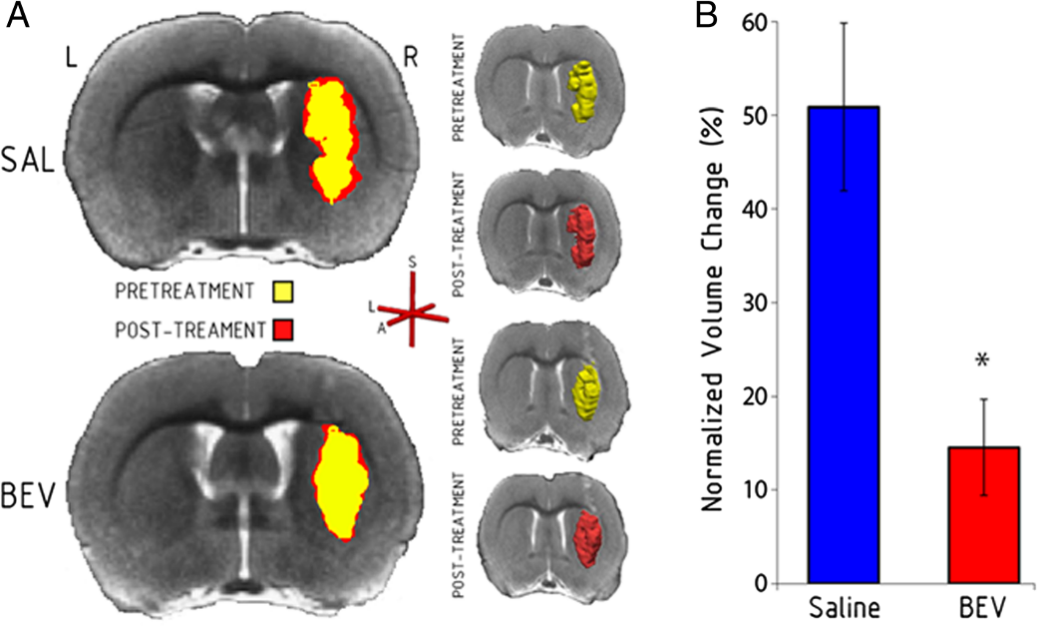
**Bevacizumab slows the growth of brain metastases.** The volume of intracerebral A549 human lung adenocarcinoma tumor was determined using T_2_-weighted MRI. **A)** Representative tumors are shown for pretreatment (yellow) and post-treatment (red) in the saline control (SAL) and bevacizumab-treated (BEV) animals. A 3-dimensional representation is shown for each tumor. The axis labels are A, anterior, L, left, and S, superior. **B)** Normalized tumor growth in saline control and bevacizumab treated animals (n = 8 per group). Data are shown as mean ± standard error of the mean change from baseline. * *P* = 0.003 versus the saline control group.

To evaluate the growth of A549 human lung adenocarcinoma xenografts in rat brain we used standard anatomical T_2_-weighted imaging (Figure 1). The tumors were characterized as a single solid mass with clearly delineated tumor borders defined between the hyperintensity of the tumor and the isointensity of normal surrounding brain on a central tumor-bisecting slice. Over the two days between pre- and post-treatment MRI, the morphology within the tumor border showed little change, with no detected increase in necrosis. Tumor growth was apparent in all saline control rats, with a 50.9 ± 8.9% increase in tumor volume (*P* = 0.011 compared to baseline). In the bevacizumab-treated rats, mean tumor volume increased by 14.6 ± 5.1% compared to baseline, although one rat showed a 5% decrease in tumor volume. Bevacizumab significantly slowed tumor growth compared to the saline controls (t_(14)_ = 3.52, *P* = 0.003). Pre and post-treatment tumor volumes are shown in Table 1.

**Table 1:** **Summary of magnetic resonance imaging results for tumor volume, longitudinal relaxivity, blood volume and passive permeability**

	Saline control		Bevacizumab treatment	
	Pre	Post	Pre	Post
Tumor volume, range (mm^3^)	7.34–36.9	9.20–58.2	6.96–46.8	8.13–44.5
Tumor volume, mean ± sd (mm^3^)	14.7 ± 9.4	22.7 ± 15.7	16.1 ± 13.2	17.8 ± 12.3
R_1_, range (ms^–1^)	0.45–0.54	0.42–0.55	0.45–0.57	0.49–0.59
R_1_, mean ± sd (ms^–1^)	0.53 ± 0.03	0.50 ± 0.04	0.53 ± 0.04	0.54 ± 0.04
Steady-state rCBV, range	0.76–2.89	0.63–1.63	0.92–1.61	0.63–1.55
Steady-state rCBV, mean ± sd	1.45 ± 0.83	1.17 ± 0.45	1.20 ± 0.26	0.93 ± 0.26
K^trans^, range (min^–1^)	0.027–0.084	0.026–0.084	0.016–0.064	0.014–0.054
K^trans^, mean ± sd (min^–1^)	0.047 ± 0.018	0.056 ± 0.018	0.043 ± 0.016	0.031 ± 0.014

R_1_: longitudinal relaxivity, rCBV: relative cerebral blood volume, K^trans^: vascular transfer coefficient.

**Figure 2:**
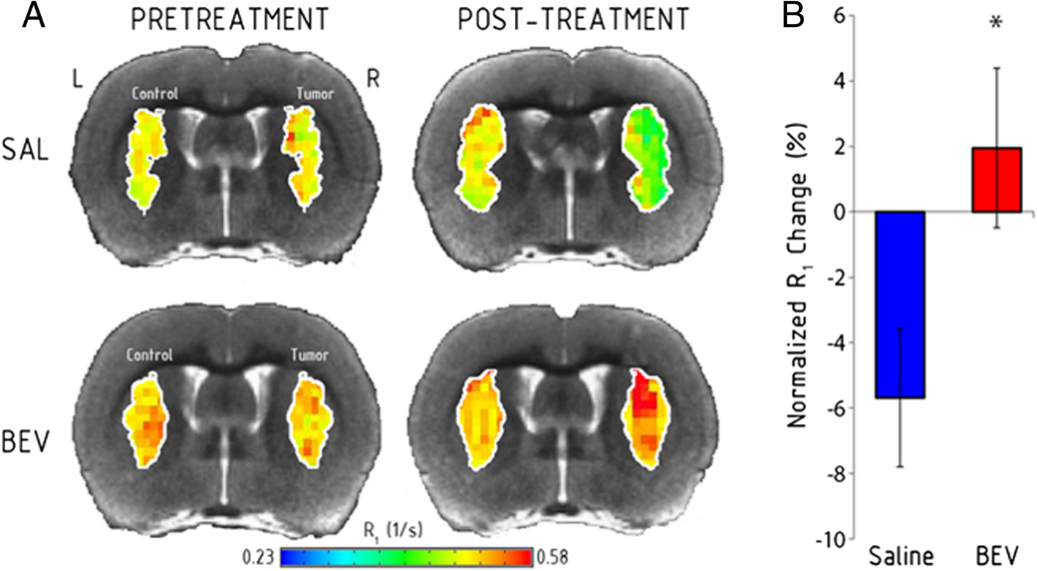
**Bevacizumab blocks the increase in edema in A549 xenografts.** The longitudinal relaxation rate (R_1_) prior to gadolinium-based contrast agent is inversely proportional to free water (edema). **A)** R_1_ maps in representative intracerebral A549 human lung adenocarcinoma xenografts (right hemisphere) compared to a matched region of interest in the control left hemisphere are shown before and after treatment with saline (SAL) or bevacizumab (BEV). **B)** Normalized change in R_1_ within the tumor region of interest in saline control and bevacizumab-treated tumors (n = 8 per group). Data are shown as mean ± standard error of the mean change from baseline. * *P* = 0.033 versus saline control group.

We quantified the effect of bevacizumab on pre-gadolinium contrast agent longitudinal relaxation rate (R_1_) values, which serve as an indirect measure of free water present in tumor, and BAT (Figure 2; Table 1). R_1_ is inversely proportional to edema. Pre-treatment R_1_ maps were evenly split between hypointense (n = 8) and hyperintense (n = 8) tumor masses relative to contralateral grey matter. Two days later, all saline control animals had an R_1_ lower than contralateral brain (−5.69 ± 2.11% mean change from baseline, paired *t*-test *P* = 0.033) indicative of increased edema with tumor progression. In contrast, bevacizumab-treated tumors continued to show heterogeneous R_1_ maps (4 hyperintense, 4 hypointense). Mean R_1_ values increased by 1.95 ± 2.43% after bevacizumab, which was not different from baseline, but was significantly different from the saline controls (t_(14)_ = −2.37, *P* = 0.033). No significant difference in average R_1_ changes was found in temporalis muscle or contralateral grey matter comparing treatment groups.

**Figure 3:**
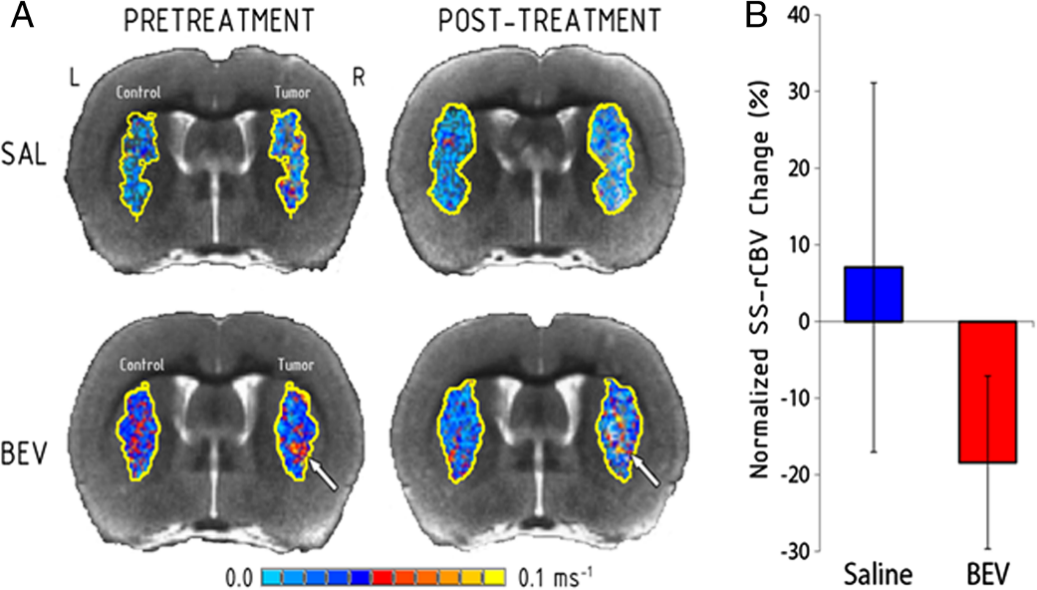
**Effect of bevacizumab on tumor blood volume.** Steady-state ΔR_2_* maps of relative cerebral blood volume (SS-rCBV) were measured in intracerebral A549 human lung adenocarcinoma xenografts. **A)** SS-rCBV in representative animals comparing control left hemisphere and tumor (right hemisphere, arrows) before and after saline (SAL) or bevacizumab (BEV). High rCBV is seen as mottled hot spots of hyperintensity. **B)** Normalized SS-CBV in tumors in the saline and bevacizumab-treatment tumors (n = 8 per group). Data are shown as mean ± standard error of the mean change from baseline.

We used high resolution steady-state ferumoxytol ΔR_2_* maps to measure tumor rCBV (Figure 3; Table 1). Mottled areas of inflated blood volume were detected within the tumor border, but overall blood volumes were not different from normal brain. Two pretreatment tumors and no post-treatment tumors showed elevated rCBV (Table 1). There was no change in rCBV with tumor growth or with bevacizumab treatment in the A549 lung cancer xenograft model.

**Figure 4:**
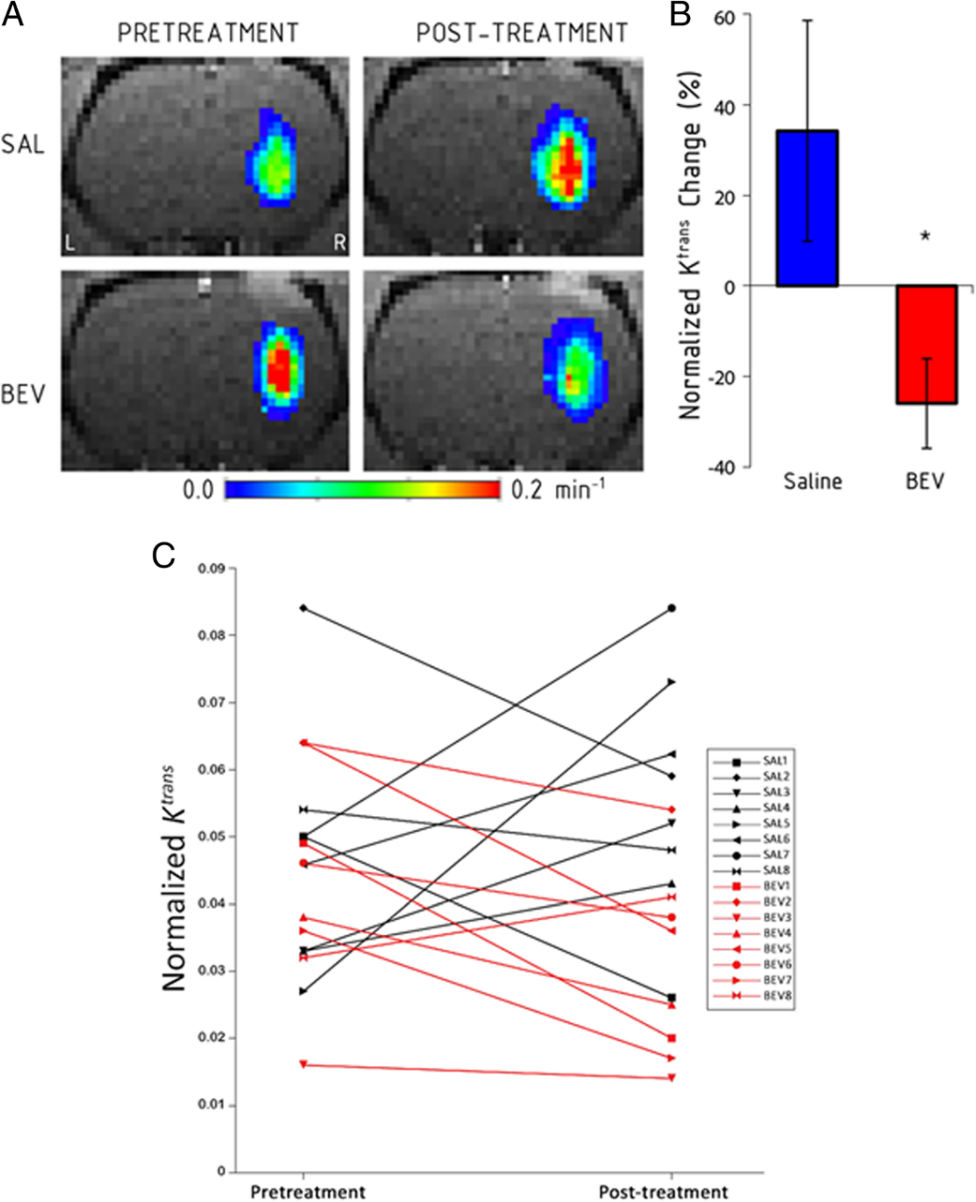
**Bevacizumab decreases tumor vessel permeability.** K^trans^ as a measure of vascular permeability was determined using dynamic contrast-enhanced MRI. **A)** Representative K^trans^ maps of A549 intracerebral xenografts before and after saline (SAL) or bevacizumab (BEV). **B)** Normalized change in K^trans^ in the saline control and bevacizumab-treated animals (n = 8 per group). Data are shown as mean ± standard error of the mean change from baseline. * *P* < 0.05 versus saline control group. **C)** Pretreatment and post-treatment K^trans^ values in individual rats in the saline (black) and bevacizumab (red) groups.

We investigated the vascular permeability of lung cancer brain metastases using DCE-MRI before and after treatment. Two-compartment model analysis yielded voxel-wise measurements of the rate transfer constant, K^trans^ and extravascular-extracellular volume fraction, v_e_. Tumors showed high permeability in the central region of the tumor on K^trans^ maps, while permeability decreased radially towards the tumor boundary (Figure 4A). Baseline K^trans^ was not different between groups (*P* = 0.65) but was inconsistent between animals, varying from 0.016 to 0.084 min^–1^ (Table 1). For the saline control group, mean K^trans^ increased by 34 ± 24% (Figure 4B) (*P* = 0.36), but this included 3 of 8 rats in which K^trans^ actually decreased with tumor progression (Figure 4C). Bevacizumab resulted in a 26 ± 10% decrease in vessel permeability compared to baseline (*P* = 0.028), which was significantly reduced compared to the saline control (t_(14)_ = 2.29, *P* = 0.04). One bevacizumab-treated rat showed increased permeability (Figure 4C). The extravascular-extracellular volume fraction, v_e_ was not different between groups and was not altered by bevacizumab treatment.

**Figure 5:**
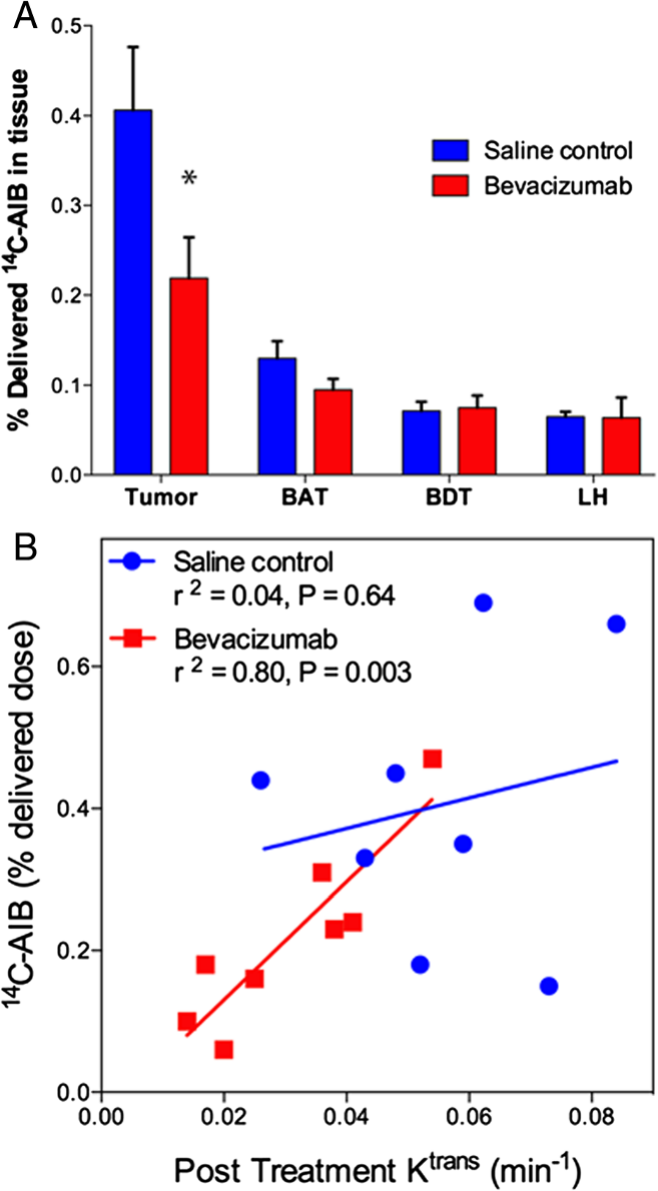
**Bevacizumab decreases AIB passive permeability. A)** The % delivered dose of ^14^C-AIB was measured in tumor, brain around tumor (BAT), brain distant from tumor (BDT) and contralateral left hemisphere (LH) in saline control and bevacizumab-treated rats with intracerebral A549 xenografts. Bevacizumab decreased AIB concentration in the tumor mass compared to saline control (* *P* = 0.04) but the effect was not significant in other brain regions. Data are indicated as mean ± SEM of % delivered dose. **B)** Correlation of AIB concentration with K^trans^ measurements of vascular permeability.

Leakage of the low molecular weight marker ^14^C-AIB was measured in dissected intracerebral tumor, BAT, BDT and contralateral left hemisphere as a surrogate for drug delivery (Figure 5A). The saline control tumor showed elevated AIB levels in the tumor mass (0.41 ± 0.07% delivered dose) and in the BAT (0.13 ± 0.02% delivered dose) compared to normal brain tissues (0.065 ± 0.005% delivered dose; *P* = 0.003 and *P* = 0.004 respectively). Bevacizumab-treated tumors also showed elevated tracer levels in tumor (0.22 ± 0.05% delivered dose) and BAT (0.10 ± 0.01% delivered dose) compared to normal brain (0.064 ± 0.022; *P* = 0.015 and *P* = 0.005 respectively). Compared with the saline control group, bevacizumab significantly decreased AIB passive permeability to the tumor mass (t_(14)_ = 2.25, *P* = 0.04). AIB levels in BAT, ipsilateral BDT and contralateral left hemisphere were not affected by bevacizumab treatment. Serum activity of ^14^C-AIB did not differ between saline control and bevacizumab (0.51 ± 0.04 vs. 0.48 ± 0.03, respectively, *P* = 0.54), nor did AIB clearance (saline control 83.4 ± 3.0%; bevacizumab 85.1 ± 2.7%, *P* = 0.68).

The post-treatment K^trans^ values were significantly correlated with AIB tracer levels within the tumor for the bevacizumab group (*r*^2^ = 0.80, *P* = 0.003, Figure 5B). In contrast, there was no correlation between post-treatment K^trans^ and the passive permeability of ^14^C-AIB in the control group (*r*^2^ = 0.04, *P* = 0.64).

## Discussion

A recent review of the future of bevacizumab treatment for glioblastoma [24] underscores two important themes that are equally valid for treatment of metastases: 1) the importance of identifying imaging markers that predict individual response; and 2) the importance of investigating bevacizumab in combination with other therapies. This current preclinical study sought to address these points by acquiring quantitative MRI parameters in conjunction with tracer passive permeability as a marker of drug delivery in a brain metastasis model.

The primary goal for using bevacizumab as a cancer therapeutic is to block the development of tumor neovasculature in order to starve the tumor to decrease tumor growth and/or kill existing tumor. We found that bevacizumab significantly slowed the growth of the rapidly growing A549 lung cancer brain metastasis model but overall did not induce tumor regression. Blocking VEGF-A with bevacizumab does not affect A549 cell proliferation in vitro [25], suggesting that the effects of bevacizumab on tumor growth in this study were not due to direct cytotoxicity. Antitumor efficacy of anti-angiogenics has been demonstrated in brain metastasis models [26, 27] and in the clinical setting [11, 13]. We did not assess long term tumor growth inhibition or survival in this study because our aim was to obtain matching AIB permeability measurements for every animal in order to correlate with early post-treatment MRI biomarkers.

Elevated blood volume, >1.75 compared to normal brain, is thought to indicate actively growing tumor, at least in high grade glioma [17, 23]. We evaluated rCBV in brain metastases using the steady-state MRI technique with ferumoxytol iron oxide nanoparticles as a blood pool contrast agent to obtain very high resolution blood volume maps [23]. We found mottled hot spots of elevated rCBV, but overall blood volume was low in the A549 model, despite the rapid growth of these tumors. The low baseline rCBV in this model limited the possibility of observing a significant effect of bevacizumab. Previous preclinical studies have demonstrated decreased blood volume after bevacizumab in a SCLC intracerebral xenograft model [28] and prostate cancer brain metastases [29], as well as glioblastoma models [17, 30].

A secondary goal for using bevacizumab in GBM is to reduce edema, but it is unclear if this mechanism will be effective in brain metastases. Cerebral metastases cause significant edema and mass effects, decreasing quality of life due to neurological deficits and headache. Corticosteroids are typically used to control edema in CNS malignancies, but their use is associated with a multitude of dose- and time-dependent adverse side effects [31]. We assessed interstitial fluid environment changes in the tumor by measuring the pre-contrast longitudinal relaxation rate on quantitative MRI [15, 16]. In contrast with normal tumor progression and concomitant edema in control animals evidenced here by a decrease in R_1_, the R_1_ was relatively stable in bevacizumab-treated animals. A possible explanation is that the treatment reduced the permeability of plasma fluid from the vascular space into the interstitial space, thereby preventing the exacerbation of edema within the tumor. Alternatively, an increase in tumor R_1_ may reflect hypocellularity and is thus a generic biomarker of early cytotoxic response [32]. Quantitative transverse relaxation measurements may reveal more detailed changes in free water content [33]. Our results support the use of bevacizumab as an effective (albeit expensive) alternative to steroid use in brain metastases.

A final rationale for the use of bevacizumab in brain metastases is the concept of vascular normalization [6, 7]. Blocking VEGF signaling in systemic tumors produces a morphologically and functionally normalized vasculature by pruning immature vessels and improving perivascular cell and basement membrane coverage and function [34]. It is hypothesized that normalization of existing tumor vasculature will improve chemotherapy delivery and chemotherapy/radiotherapy efficacy [7, 24, 34]. Brain metastases can induce neovascularization, with leaky vessels, but can also co-opt existing brain vasculature, with near-normal BBB, particularly in the tumor-infiltrated BAT [35]. Clinically brain metastases can show highly variable permeability, and this is recapitulated in hematogenous metastases in animal models [36]. Xenograft models typically provide more uniform permeability, given similar size and location of tumor mass. However, permeability was highly variable in the A549 xenograft model, with nearly one order of magnitude variability in K^trans^ values among the different tumors at baseline. Variable permeability may reflect inconsistent neovascularization versus vessel co-option with resulting differences in BBB dysfunction.

We hypothesize that vascular normalization with anti-VEGF agents may restore the low permeability characteristics of the BBB in brain tumors, therefore actually decreasing drug delivery [22]. Several reports support the concept that vascular normalization with anti-VEGF agents may decrease BTB permeability. The VEGF receptor inhibitor vandetanib decreased temozolomide-induced apoptosis in brain tumor models [37], while another anti-VEGF receptor agent cediranib decreased the permeability of the BBB in a prostate cancer brain metastasis xenograft model [29]. In the current study, permeability in the A549 tumor mass and tumor perimeter decreased following bevacizumab, which correlated with decreased passive permeability of ^14^C-AIB. AIB was chosen because it is a low molecular weight marker (103 Da) equivalent in size to many chemotherapeutics, and because it is not a substrate for the P-glycoprotein family of drug exporters, so its concentration in tumors is primarily a function of vascular permeability. One limitation of the study was that the gross dissection technique used for AIB quantification localized inhomogeneities that could be better detected with quantitative autoradiography. Nevertheless, AIB levels were was highly correlated with K^trans^ after bevacizumab treatment, but not in the saline controls, suggesting that VEGF blockade did indeed induce BBB normalization in this metastasis model. Given these results, it is reasonable to expect that bevacizumab treatment may result in decreased delivery and resultant intratumoral concentration of chemotherapeutic agents whose molecular weights are similar to AIB, thereby decreasing therapeutic effects. Additionally, vascular normalization may decrease contrast leakage on MRI to give the appearance of response to therapy, while asymptomatic tumor growth proceeds masked by an intact BBB. This phenomenon, known as pseudoresponse, has been documented in glioblastoma [38] and may also occur in brain metastases treated with bevacizumab.

## Conclusions

This study used MRI biomarkers and delivery of a low molecular weight radioactive tracer to assess the effects of bevacizumab on the vasculature in and around intracerebral human lung cancer xenografts and the permeability of the BTB to AIB after bevacizumab treatment. Compared to saline controls, bevacizumab slowed tumor growth, blocked the increase in edema, and significantly decreased both tumor vessel permeability and the potential for drug delivery. These data support the efficacy of MRI measurement to clarify the effects of vascular targeting agents in brain metastases, and provides compelling evidence of bevacizumab-mediated vascular normalization. The current study underscores the importance of determining the vascular normalization window based on such factors as tumor type, bevacizumab dose and timing, and initial permeability state.

## Abbreviations

AIB: Aminoisobutyric acid
BAT: Brain around tumor
BBB: Blood–brain barrier
BDT: Brain distant to tumor
CNS: Central nervous system
DCE: Dynamic contrast enhanced
FA: Flip angle
FLASH: Fast low-angle shot
FOV: Field of view
MRI: Magnetic resonance imaging
RCBV: Relative cerebral blood volume
TE: Echo time
TI: Inversion time
TR: Repetition time
LH: Left hemisphere
NSCLC: Non-small cell lung cancer
RARE: Rapid acquisition refocusing echoes
ROI: Region of interest
SS-rCBV: Steady-state relative cerebral blood volume
VEGF: Vascular endothelial growth factor.
